# Is There a Survival Benefit of Adjuvant Chemotherapy in Stage IC1 Epithelial Ovarian Cancer Patients? A Meta-Analysis

**DOI:** 10.3390/curroncol29080454

**Published:** 2022-08-15

**Authors:** Vasilios Pergialiotis, Efstathia Liatsou, Aggeliki Rouvali, Dimitrios Haidopoulos, Dimitrios Efthymios, Michalis Liontos, Alexandros Rodolakis, Nikolaos Thomakos

**Affiliations:** 1 1st Department of Obstetrics and Gynecology, Division of Gynecologic Oncology, Alexandra Hospital, National and Kapodistrian University of Athens, 10679 Athens, Greece; 2 Department of Clinical Therapeutics, Division of Oncology, Alexandra Hospital, National and Kapodistrian University of Athens, 10679 Athens, Greece

**Keywords:** ovarian cancer, cyst rupture, stage IC1, survival, meta-analysis

## Abstract

The purpose of the present systematic review is to clarify whether adjuvant chemotherapy improves survival rates in women with stage IC1 ovarian cancer. We searched Medline, Scopus, Clinicaltrials.gov, EMBASE, Cochrane Central Register of Controlled Trials CENTRAL and Google Scholar. We considered comparative observational studies and randomized trials that investigated survival outcomes (progression-free (PFS) and overall survival (OS)) among women with intraoperative rupture of early-stage epithelial ovarian cancer who received adjuvant chemotherapy and those that did not. Eleven studies, which recruited 7556 patients, were included. The risk of bias was defined as moderate after assessment with the Risk of Bias in non-Randomized Trials tool. Meta-analysis was performed with RStudio. Seven studies investigated the impact of adjuvant chemotherapy on recurrence-free survival of patients experiencing intraoperative cyst rupture for otherwise stage I ovarian cancer. The outcome was not affected by the use of adjuvant chemotherapy as the effect estimate was not significant (HR 1.24, 95% CI 0.74, 2.04). The analysis of data from 5 studies similarly revealed that overall survival rates were comparable among the two groups (HR 0.75, 95% CI 0.54, 1.05). This meta-analysis did not detect any benefit from adjuvant chemotherapy for stage IC ovarian cancer patients with cyst rupture. However, conclusions from this investigation are limited by a study population which included multiple histologic subtypes, high and low grade tumors and incompletely staged patients.

## 1. Introduction

Ovarian cancer is the leading cause of death among gynecologic cancers despite the fact that its prevalence declined during the last four decades [1]. The lifetime risk of developing ovarian cancer is about 1 in 78, and approximately 21,000 receive a new diagnosis of the disease in the United States yearly, and ultimately, 13,700 succumb to it each year, with an expected 5-year relative survival rate of 49% [2]. Unfortunately, most patients have metastatic disease at the time of diagnosis as the identification and diagnosis of early stage ovarian cancer is challenging.

Outcomes for low grade, early stage ovarian cancer with no extra-capsular growth and with negative peritoneal cytology are excellent with expected survival rates exceeding 90%. In these cases, survival rates are expected to exceed 90%. However, in the presence of intraoperative rupture of the ovarian capsule both progression-free (PFS) as well as overall survival (OS) rates are expected to fall. In a recent meta-analysis that was based on 17 studies, researchers observed that cyst rupture was associated with a nearly double risk of recurrence (hazards ratio 1.92, 95% CI 1.34–2.76) and a 48% increased chance of death (hazards ratio 1.48, 95% CI 1.15–1.91) compared to women that had intact cyst removal [3].

The most prominent studies that addressed the impact of adjuvant chemotherapy in stage I disease are the International Collaborative Ovarian Neoplasm 1 [ICON1] and Adjuvant ChemoTherapy In Ovarian Neoplasm [ACTION] trials, which were conducted in the same period and depicted a clear survival benefit both in terms of disease-free as well as overall survival rates [4]. Current guidelines support the use of adjuvant chemotherapy in women with FIGO stage IC disease [5,6,7]. These are driven from large meta-analyses that support its use in stage I patients [8,9]. However, a growing body of evidence supports the lack of survival benefit in the subgroup of patients with early-stage disease that experience intraoperative cyst rupture. In a large population-based study, Matsuo et al. reported the absence of a survival benefit in these women [10]. However, the study was limited by the absence of appropriate staging of included patients and the lack of reporting of the grade of differentiation in serous and endometrioid tumors.

The aim of the present meta-analysis is to gather evidence from published observational and randomized controlled trials in order to evaluate the impact of adjuvant chemotherapy on survival rates of women with early-stage ovarian cancer that experience intraoperative tumor spillage (stage IC1 disease) as well as to assess the methodological quality of studies published in this field. Current recommendations suggest that appropriately staged low-grade early stage disease may be treated with expectant management compared to high-grade early stage disease, in which case patients should be offered adjuvant chemotherapy [6]. The aim of the present meta-analysis is to gather evidence from published observational and randomized controlled trials in order to evaluate the impact of adjuvant chemotherapy on survival rates of women with early-stage ovarian cancer that experience intraoperative tumor spillage (stage IC1 disease) as well as to assess the methodological quality of studies published in this field.

## 2. Materials and Methods

### 2.1. Protocol and Registration

The present meta-analysis was designed according to the Preferred Reporting Items for Systematic Reviews and Meta-Analyses (PRISMA) guidelines [11]. The study was based on aggregated data that have already been published in the international literature. Patient consent and institutional review board approval were not retrieved as they are not required in this type of study. The study’s protocol was published in the International Prospective Register of systematic reviews (PROSPERO) prior to the conduct of this review (registration number: CRD42021290985).

### 2.2. Types of Studies and Patients

Eligibility criteria for the inclusion of studies were predetermined. Randomized trials and observational studies (prospective and retrospective) that reported survival outcomes (progression-free and overall survival rates) among women with stage IC1 ovarian cancer that received adjuvant chemotherapy and those that did not were considered eligible for inclusion. Studies were included irrespective of the chemotherapy scheme that was used. Included schemes were expected to vary; however, given that the majority of studies use platinum-based compounds in accordance to current guidelines, a pre-requisite for inclusion was the use of a platinum compound with or without the addition of a taxane or in the setting of a FOLFOX scheme (oxaliplatin, leucovorin and 5-FU) for mucinous ovarian carcinomas. Sub-analysis was initially designed in case of significant differences among studies in terms of this variable. Studies that reported survival outcomes of patients suffering from non-epithelial ovarian cancer were excluded from the present study.

### 2.3. Information Sources and Search Methods

Two authors (V.P and E.L.) searched Medline (1966–2021), Scopus (2004–2021), Clinicaltrials.gov (2008–2021), EMBASE (1980–2021), Cochrane Central Register of Controlled Trials CENTRAL (1999–2021) and Google Scholar (2004–2021), along with the reference lists of electronically retrieved full-text papers. The date of the last search was set at December, 2021. The Rayyan web app was used for the screening process [12]. Any disagreements between reviewers were resolved by a third reviewer (M.L.). The search strategy included the text words “ovarian cancer; spillage; rupture; early stage; IC1” and is presented in brief in the Appendix.

Studies were selected in three consecutive stages. Following deduplication, the titles and abstracts of all electronic articles were independently screened by two authors (V.P and E.L) to assess their eligibility. The decision for inclusion of studies in the present meta-analysis was taken after retrieving and reviewing the full version of articles that were considered potentially eligible. Discrepancies that arose in this latter stage were resolved by consensus from all authors.

### 2.4. Predefined Outcomes

Outcome measures were predefined during the design of the present systematic review. Data extraction was performed using a modified data form that was based in Cochrane’s data collection form for intervention reviews for RCTs and non-RCTs [13].

Differences in progression-free and overall survival rates among women with stage IC1 disease that were treated with adjuvant chemotherapy compared to those that were treated with expectant management were pre-defined as the main outcomes of this study. We also sought to retrieve side-effects and report differences in significant toxicity among the two groups.

### 2.5. Assessment of Risk of Bias and Quality of Evidence

The methodological quality of included randomized controlled trials was initially designed to be assessed by two independent reviewers (E.L and D.H) using the Risk of Bias 2 (RoB 2) tool. The quality of non-randomized trials was assessed with Risk of Bias in non-Randomized Trials (ROBINS-I) tool which incorporates 5 domains that investigated bias that arises (i) from confounders, (ii) from selection of participants, (iii) from selective reporting in intervention measures, (iv) from deviations from intended interventions, (v) due to missing data, (vi) from selective reporting in outcome measures and vii) from selective reporting of outcomes.

### 2.6. Statistical Analysis

Statistical meta-analysis was performed with RStudio using the meta and metafor functions (RStudio Team (2015). RStudio: Integrated Development for R. RStudio, Inc., Boston, MA URL http://www.rstudio.com/ (accessed on 1 December 2021). Statistical heterogeneity was not considered during the evaluation of the appropriate model of statistical analysis as the anticipated methodological heterogeneity of included studies did not leave space for assumption of comparable effect sizes among studies included in the meta-analysis [14]. Confidence intervals were set at 95%. We calculated pooled hazard ratios (HR) and 95% confidence intervals (CI) with the Hartung–Knapp–Sidik–Jonkman model instead of the traditional Dersimonian–Laird random effects model analysis (REM). The decision to proceed with this type of analysis was taken after taking into consideration recent reports that support its superiority compared to the Dersimonian–Laird model when comparing studies of varying sample sizes and between-study heterogeneity [15]. When variables where expressed as median (range), median (interquartile range) or interquartile range and sample size, transformations were performed to acquire the mean and standard deviation to include the studies in the meta-analysis [16].

A cut-off of at least 10 studies per investigated outcome was pre-defined as mandatory for the investigation of publication bias with visual inspection of funnel plots and evaluation of the results of Egger’s regression and Begg–Mazumdar’s rank correlation tests. 

The potential presence of small-study effects was evaluated with Rücker’s Limit meta-analysis and the possibility of p-hacking was also investigated with inspection of the results of the p-curve analysis.

### 2.7. Prediction Intervals

Prediction intervals (PI) were also calculated, using the meta function in RStudio, to evaluate the estimated effect that is expected to be seen by future studies in the field. The estimation of prediction intervals takes into account the inter-study variation of the results and express the existing heterogeneity at the same scale as the examined outcome. 

### 2.8. Subgroup Analysis

Subgroup analysis of primary outcomes was initially designed to be accomplished taking into consideration the use of taxane or not along with the use of platinum derivatives and the methodology that was used during the conduct of each study (RCT/non-RCT). However, the lack of differences in these variables rendered impossible this type of analysis. 

## 3. Results

**Figure 1:**
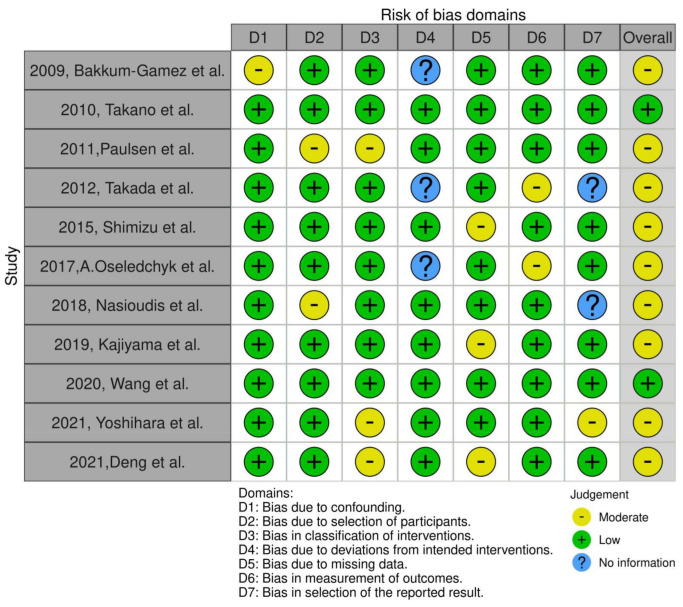
ROBINS-I evaluation of included studies [17,18,19,20,21,22,23,24,25,26,27].

We identified 11 studies relevant to the subject matter [10,17,18,19,20,21,22,23,24,25,26,27]. Of those, we chose to omit the largest one which was population-based and discuss it separately as its size would overshadow the effect of smaller studies [10]. Overall, 11 retrospective cohort studies involving 7556 patients were included in the present systematic review. Their methodological characteristics are summarized in Table 1. Primary endpoints of each study significantly differed, designating gross heterogeneity in outcome reporting. Underreporting in rates of fertility-sparing surgery and presence of ascites was denoted. Survival outcomes for patients receiving fertility-sparing surgery were not available. Study evaluation with the ROBINS-I tool revealed the presence of moderate risk of bias in the majority of studies Figure 1.

**Figure 2:**
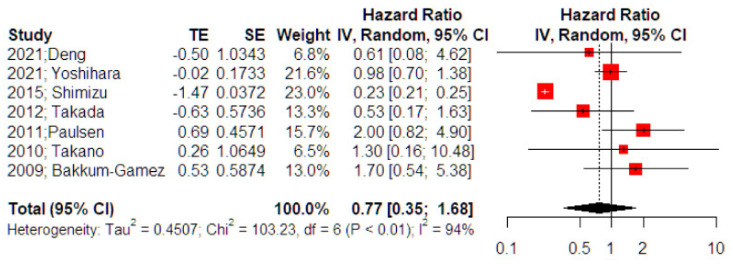
Forest plot of recurrence-free survival among patients with intraoperative cyst rupture that receive chemotherapy compared to those treated with expectant management (right side): Vertical line = “no difference” point between the two groups. Red squares = odds (hazard) ratios; diamond = pooled mean odds (hazard)radio and 95% CI for all studies; horizontal black lines = 95% CI [17,18,23,24,25,26,27].

Seven studies investigated the impact of adjuvant chemotherapy on recurrence-free survival of patients experiencing intraoperative cyst rupture for otherwise stage I ovarian cancer. The outcome was not affected by the use of adjuvant chemotherapy as the effect estimate was not significant (HR 0.77, 95% CI 0.35, 1.68) (Figure 2). Following the influence diagnostics analysis, we observed that the study of Shimizu et al. served as a potential outlier. However, even after its removal the effect remained non-significant (HR 1.24, 95% CI 0.74, 2.04). Leave-one-out meta-analysis was also performed to evaluate the independent effect of each study on the significance of the overall effect size and did not alter the significance level. Evaluation of the result for small study effects revealed a significant reduction in the risk of death among patients that received adjuvant chemotherapy (HR 0.62, 95% CI 0.57, 0.67); however, the small number of included studies and the residual heterogeneity that was attributed to factors beyond small-study effects (*p* < 0.001) renders this latter finding unreliable. 

**Figure 3:**
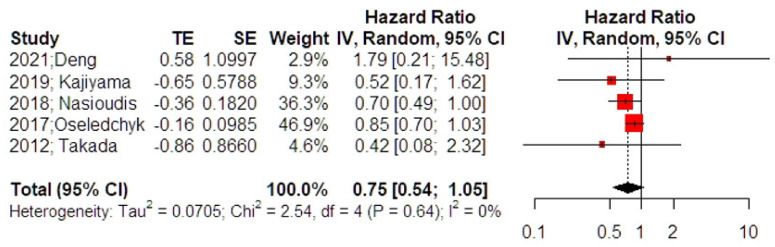
Forest plot of overall survival among patients with intraoperative cyst rupture that received chemotherapy compared to those treated with expectant management (right side): Vertical line = “no difference” point between the two groups. Red squares = odds (hazard) ratios; diamond = pooled mean odds (hazard)radio and 95% CI for all studies; horizontal black lines = 95% CI [17,20,21,22,24].

The meta-analysis of 5 studies revealed that the overall survival rates of patients with intraoperative cyst rupture were not affected using adjuvant chemotherapy (HR 0.75, 95% CI 0.54, 1.05) (Figure 3); however, the result was close to the level of statistical significance (*p* = 0.076). Analysis for outliers and influence diagnostics did not distinguish a study that could potentially alter the overall effect estimate. Leave-one-out meta-analysis also did not alter the significance of the primary effect size. Adjustment for small study effects revealed, however, a significant effect estimate that pointed towards a reduced overall survival among women that did not receive adjuvant chemotherapy (HR 0.76, 95% CI 0.59, 0.98). Despite the small number of included studies, residual heterogeneity beyond small-study effects was insignificant (*p* = 0.546), pointing towards a potential evidential value. 

## 4. Discussion

The findings of our study suggest that adjuvant chemotherapy may overtreat women with early-stage epithelial ovarian cancer that is ruptured during surgery. However, these findings are based in retrospective cohorts that have an inherent degree of bias. A potentially significant treatment effect of adjuvant chemotherapy, in terms of overall survival, was observed following sensitivity analysis; however, this should be reviewed with caution due to the small number of studies included in this analysis.

A recent meta-analysis based on 17 retrospective cohort studies et al. highlighted the negative impact of intraoperative ovarian capsule rupture on progression-free survival and overall survival of women with early-stage disease [3]. Specifically, the authors reported a nearly double risk of experiencing a recurrence (HR 1.92, 95% CI 1.34, 2.76) and a 50% increase in the risk of dying (HR 1.48, 95% CI 1.15, 1.91). In 2013, Kim et al. supported this risk by conducting a meta-analysis that was based on nine studies, most of which were also referenced in latest meta-analysis that was previously mentioned [28]. They also mentioned that the risk of recurrence was comparable among patients experiencing intraoperative cyst rupture and those that did not when complete surgical staging was undertaken and adjuvant platinum chemotherapy was administered (HR 1.49, 95% CI 0.45, 4.95, results based on data from two studies). Taking this information into account, it was indirectly assumed that adjuvant chemotherapy may be necessary for women with stage IC1 epithelial ovarian cancer as it could help prevent recurrences owing to the rupture of the tumor. 

In contrast lies the largest series published to date by Matsuo et al., who reported a prevalence of intraoperative rupture of 47.7%, with younger age at surgery being an independent predictive factor of experiencing this complication [10]. The proportion of patients that ultimately received chemotherapy was 78.1%. Of note, none of the histologic subgroups of patients had a clear survival benefit as the authors reported (adjusted HR 0.86, 95% CI 0.56–1.31 for clear-cell; adjusted HR 1.08, 95% CI 0.42–2.74 for serous; adjusted HR 1.11, 95% CI 0.55–2.27 for mucinous; and adjusted HR 2.81, 95% CI 0.85–9.30 for endometrioid subtype).

The importance of adjuvant chemotherapy in early-stage ovarian cancer is an important topic that has been extensively discussed in the international literature as it often involves women of reproductive age with a strong desire for fertility preservation. Lawrie et al. observed in a systematic review that was conducted with the Cochrane Collaboration that adjuvant chemotherapy can prolong survival rates in patients with FIGO stage I/IIa [9]. However, questions about the actual impact of several parameters exist. For instance, evidence drawn from the Cochrane review suggests that disease-specific survival in subgroups of cases that are optimally staged, and those that are not seem to be comparable irrespective of the use of adjuvant chemotherapy. Furthermore, despite the survival outcomes reported by Matsuo et al. it remains well known that the chemotherapy response in patients with clear-cell and mucinous subtypes is significantly reduced, therefore rendering extrapolation of the results extremely problematic in the sum of early-stage cases [29,30].

### 4.1. Strengths and Weaknesses of the Study

Our study is based on a meticulous review of the literature that aimed to reduce the possibility for potential article losses. Several sensitivity analyses were performed with the use of advanced meta-analytical models that aim to investigate the influence of potential bias on the statistical significance of the overall effect estimate. 

Nevertheless, it should be emphasized that the retrospective nature of the included studies predisposes them to an inherent possibility of bias, and, following their evaluation, we observed that this effect seemed to be of moderate importance. 

Several factors might affect the findings of our study with histological subtype and grade of differentiation of included cases being the most predominant. On this point we must note that most of the evidence published in the international literature is mainly derived from cases with papillary serous carcinoma, which is the predominant subtype in the majority of cases. In the presented series, serous histology accounted for only a small proportion of cases, rendering extrapolation of our findings difficult in the clinical setting. However, given the findings of the population-based study of Matsuo et al., one may assume that the importance of this latter factor on survival outcomes of stage IC1 might not be as significant as hypothesized. In another study conducted by the Gynecologic Oncology Group, researchers reported that in early stage ovarian cancer the use of six cycles, rather than three does not benefit women with histologic subtypes, other than high grade serous, which could also indirectly support the findings of the study of Matsuo et al. [31]. A potentially important issue that was not addressed in this study was the grade of differentiation of the included serous and endometrioid cases. However, it should be noted that limited evidence concerning the impact of grade as a potential predictor of survival argues the fact that it can significantly affect prognosis in stage IC1 patients [17,27]. The extent of staging remains also questionable in several studies as does the cytology status of peritoneal washings, as indicated in Table 1, thus pointing the need for further research in this field. 

### 4.2. Implications for Clinical Practice and Future Research

Given that most cases with stage IC1 epithelial ovarian cancer are women of reproductive age that wish to preserve their fertility, it becomes easily understandable that the use of adjuvant chemotherapy in the absence of a clear survival benefit should potentially be regarded as overtreatment. On the other hand, the clinical decision to leave these women unprotected from the risk of recurrence raises several ethical concerns in the absence of robust evidence. 

The findings of this meta-analysis suggest that adjuvant chemotherapy may not be beneficial in terms of survival outcomes in stage IC1 ovarian cancer patients. However, several factors prohibit firm conclusions as most of the evidence is drawn from studies that involve populations that are treated both optimally and suboptimally as well as the inclusion of several histologic subtypes involving high- and low-grade tumors, an observation which could also indirectly support the findings for non-serous subtypes in the study of Matsuo. It should also be mentioned that the information provided is based on cases that involve clear-cell (53%) and endometrioid subtypes (43%), therefore rendering evidence about serous and mucinous tumors (which accounted for 0.5% and 2.8% of cases, respectively) very limited. Therefore, although until further research becomes available, the omission of adjuvant chemotherapy is unavoidable, at least in high-grade cases as well as those that are suboptimally staged. A potential consideration would be to inform patients about the pros and cons of chemotherapy in appropriately staged low-grade cases that experience intraoperative cyst rupture, taking into consideration the aforementioned limitations and balancing them with the fertility potential and desire. 

Given these considerations, it becomes understandable that future research is necessary to provide robust evidence and that phase III clinical trials should be designed to investigate the need for adjuvant chemotherapy in stage IC1 epithelial ovarian cancer patients after subgrouping optimally staged patients according to the histological subtype, tumor grade, menopausal status and tumor size.

## Figures and Tables

**Table 1:** Study, tumor and treatment characteristics.

Reference	Histology (%)	Staging (Yes)	Ascites (Yes)	FertilitySparing Surgery	Follow-Up (Adjuvant)	Follow-Up (Control)	Primary Endpoint
[27]	Serous (18) Clear-cell (22) Mucinous (17) Endometrioid (37) Others (6)	161/161 *	-	-	44.7 months (0.2–185)		Impact of intraoperative capsule rupture on survival outcomes
[26]	Clear-cell (100)	145/219 *	79/219	-	48 months (7–160)	43 months (3–93)	Impact of chemotherapy on survival outcomes of stage I clear-cell ovarian carcinoma patients
[25]	Serous (28) Mucinous (23) Endometrioid (23) Clear-cell (19) Others (7)	139/279	82/279	-	56 months (0–56)		Impact of chemotherapy on survival outcomes of stage I epithelial ovarian carcinoma patients
[24]	Clear-cell (100)	73/73 **	-	0/73	30 months (14–113)	56 months (13–119)	Impact of chemotherapy on survival outcomes of stage I clear-cell ovarian carcinoma patients
[23]	Serous (17) Mucinous (24) Endometrioid (17) Clear-cell (40) Others (2)	156/267 **	59/267	25/267	65 months (7–209)		Impact of chemotherapy on survival outcomes of stage I epithelial ovarian carcinoma patients
[22]	Endometrioid (64) Clear-cell (36)	1460/3552 639/1995	-	-	65 months (0–167)		Impact of chemotherapy on survival outcomes of stage I endometrioid or clear-cell ovarian carcinoma patients
[21]	Clear-cell (100)	2325/2325	-	-	59.1 months (1.1–151.4)	68.3 months (1.7–151.8)	Investigate the patterns of use and impact of chemotherapy in survival outcomes of stage I clear-cell ovarian carcinoma patients
[20]	Mucinous (100)	194/194 *	44/194	41/149	67.6 months (2.0–248.1)		Investigate the significance of the capsule status in early-stage patients with mucinous epithelial ovarian carcinoma
[19]	Clear-cell (100)	96/102	-	-	40.5 months, range (3–212)		Survival rates among different substages of stage I clear-cell ovarian carcinoma, and impact of chemotherapy
[18]	Serous (10.2) Mucinous (30.6) Clear-cell (30.6) Endometrioid (26.1) Others (2.4)	147/289 *	27/289	42/289	NA		Impact of incomplete surgery and adjuvant chemotherapy on survival rates of stage Ic1 epithelial ovarian carcinoma patients
[17]	Serous (32.9) Mucinous (31.4) Endometrioid (13.6) Clear-cell (20) Mix (2.1)	125/140 *	9/140	21/140	34 months (6–203)		Impact of adjuvant chemotherapy on survival outcomes of stage Ic1 epithelial ovarian carcinoma patients

* = comprehensive staging was defined as collection of cytologic washings, hysterectomy (if uterus present), bilateral salpingo-oophorectomy, bilateral pelvic and para-aortic lymphadenectomy, omentectomy, and peritoneal biopsies); ** = comprehensive staging same as previous without cytologic washing.

## Data Availability

All data generated or analyzed during this study are included in this article. Further enquiries can be directed to the corresponding author.
